# Potential Anti-Aging Substances Derived from Seaweeds

**DOI:** 10.3390/md18110564

**Published:** 2020-11-18

**Authors:** Lei Cao, Sang Gil Lee, Kwon Taek Lim, Hyeung-Rak Kim

**Affiliations:** 1 Institute of Marine Life Sciences, Pukyong National University, Busan 48513, Korea; caolei0630@pknu.ac.kr; 2 Department of Food Science and Nutrition, Pukyong National University, Busan 48513, Korea; sglee1125@pknu.ac.kr; 3 Department of Display Science and Engineering, Pukyong National University, Busan 48513, Korea; ktlim@pknu.ac.kr

**Keywords:** anti-aging, senescence, macroalgae, seaweeds, bioactive

## Abstract

Aging is a major risk factor for many chronic diseases, such as cancer, cardiovascular disease, and diabetes. The exact mechanisms underlying the aging process are not fully elucidated. However, a growing body of evidence suggests that several pathways, such as sirtuin, AMP-activated protein kinase, insulin-like growth factor, autophagy, and nuclear factor erythroid 2-related factor 2 play critical roles in regulating aging. Furthermore, genetic or dietary interventions of these pathways can extend lifespan by delaying the aging process. Seaweeds are a food source rich in many nutrients, including fibers, polyunsaturated fatty acids, vitamins, minerals, and other bioactive compounds. The health benefits of seaweeds include, but are not limited to, antioxidant, anti-inflammatory, and anti-obese activities. Interestingly, a body of studies shows that some seaweed-derived extracts or isolated compounds, can modulate these aging-regulating pathways or even extend lifespans of various animal models. However, few such studies have been conducted on higher animals or even humans. In this review, we focused on potential anti-aging bioactive substances in seaweeds that have been studied in cells and animals mainly based on their anti-aging cellular and molecular mechanisms.

## 1. Introduction

### 1.1. Aging and Cellular Senescence

Aging is progressive deterioration of organ structure and decline or loss of organ function, leading to the death of an organism. Several factors such as genes, nutrients, and environment are known to contribute to the aging process [1]. Calorie restriction, the most successful anti-aging regime, has been studied for almost a century (since the 1930s dietary restriction study) [2]. It is so far the most effective approach for lifespan extension and therefore interest in studying the mechanisms underlying caloric restriction has grown. The major signaling pathways involved in this process include the silent mating type information regulation 2 homolog (SIRT), AMP-activated protein kinase (AMPK), autophagy, insulin/insulin-like growth factor (IGF), and nuclear factor erythroid 2-related factor 2 (NRF2) pathways [3]. Certain bioactive compounds, especially phytochemicals, have also been studied for their anti-aging functions by regulating these pathways [4]. Of note, sex dimorphism in response to lifespan-extension interventions has been observed across species. For example, reduced IGF-1 signaling or calorie restriction have showed more profound longevity enhancement effect on females than males [5,6,7].

Aging can also occur in single cells within an organism, i.e., cellular senescence, a hallmark of aging [8]. Senescent cells lose the capacity of cell division, but they are still alive and metabolically active. In mammals, senescent cells accumulate with age and at sites of certain age-related pathologies, such as cataracts, obesity, and type 2 diabetes [9].

Senescence is usually categorized as replicative and stress-induced senescence. Cells lose a part of their telomere during each cell division. After a certain number of replications, a few telomeres of the cells will be uncapped, and those cells will undergo growth arrest, leading to replicative senescence [10]. Besides replication, various stress factors such as oncogenic and oxidative stresses can also induce genomic DNA damage, leading to permanent cell cycle arrest and consequently stress-induced senescence [11,12].

Senescence is considered a protective program, as it puts cells that are at a risk of neoplastic transformation into permanent cell cycle arrest [13]. Under normal conditions, senescent cells can be recognized and removed by the body’s immune system. However, accumulative senescent cells, which cannot be eliminated by the immune system in time, arise from multiple mechanisms. Additionally, senescent cells produce the senescence-associated secretory phenotype (SASP), which contains pro-inflammatory and matrix-degrading molecules. Through SASP, lingering senescent cells make aged tissues less functional and more vulnerable to further deterioration when faced with additional stressors [13].

### 1.2. Nutritional Benefits of Seaweeds

The algae are a heterogeneous group of aquatic, mainly eukaryotic organisms that have photosynthetic ability. Morphologically, the algae can be classified into two groups, viz., macroalgae (which are multicellular algae lacking true roots, stems and leaves) and microalgae (which are unicellular and microscopic). The marine macroalgae (seaweeds), which are the focus of this review, are generally divided into three large groups, namely, green algae (Chlorophyta), red algae (Rhodophyta), and brown algae (Ochrophyta). The seaweeds are distributed in the intertidal and photic sublittoral zones attached to rocks. The marine green algae are frequent in warmer seas and are the forerunners of land plants. The red algae have a unique phycoerythrin pigment, which usually imparts them the red color. Among the brown algae are the largest seaweeds of the world, the kelps, that look like higher plants with their roots, stems and leaves [11]. 

Seaweeds are a rich source of several essential nutrients such as dietary fibers, proteins, vitamins, and minerals [14]. They are also rich in polyunsaturated fatty acids such as eicosapentaenoic acid (EPA) and docosahexaenoic acid (DHA), which make seaweed an excellent source of vegan omega-3 fatty acids [15]. The health benefits of dietary seaweed consumption have been shown in humans. It has been reported that seaweed administration via maize- or wheat-based means to human subjects can increase their iron absorption content due to high iron content in seaweed [16]. Another epidemiology study reported that dietary seaweed consumption might decrease the risk of diabetes in men [17].

Among the marine algae, brown algae are a rich source of health-beneficial compounds. The major bioactive compounds are produced as secondary metabolites and are identified as phlorotannins, meroterpenoids, phytosterols, bisnorditerpenes, glycolipids, and pheophytin [18]. For example, phlorotannins, the oligomers of phloroglucinol, have been widely studied for their health benefits [19,20,21,22,23,24]. Ecklonia species, including *E. cava*, *E. stolonifera*, *E. bicyclis*, and *E. kurome*, are known to have diverse phlorotannins [22]. The biological activities of phlorotannins include antioxidant [25,26,27,28,29,30,31], hepatoprotective [32,33,34,35,36], anti-cancer [35,37,38], anti-obesity [39,40,41,42], anti-inflammatory [25,43,44,45,46,47,48,49], anti-diabetic [50,51,52,53], anti-skin aging [31,54,55], and skin hypopigmentation activities [56,57,58].

The utilization of algal substances in cosmeceuticals have been discussed lately, including applications on anti-skin aging, skin whitening, and anti-skin cancer [59]. Our review covered the anti-aging potential of seaweed derived substances on not only skin aging but other cell and tissue models. Through reviewing the effects of marine macroalgal substances on life extension and longevity regulating pathways, we want to explore the potential and value of seaweed-derived substances in anti-aging research and pharmaceutical application.

## 2. Lifespan Extension and Cellular Senescence Inhibition Effects of Seaweed Derivatives

The lifespan extension effects of marine macroalgal compounds have been observed in animal models, such as yeasts and flies. Fucoxanthin, a carotenoid and a major photosynthetic pigment extracted from brown algae, exhibited lifespan extension effect in both *Drosophila melanogaster* and *Caenorhabditis elegans* [60]. Porphyran is a sulfated polysaccharide from *Porphyra haitanensis*, a species of red algae. The lifespan extension effect of degraded porphyran and natural porphyran have been reported in *Drosophila* [61]. In addition to lifespan, the vitality of middle-aged flies has also been found to increase by porphyrans [61]. These findings suggest that porphyran can increase lifespan and health span, at least in *Drosophila*. Similarly, polysaccharides from the brown alga, *Saccharina japonica*, can also significantly prolong the average lifespan of both male and female *Drosophila*. However, the increase was more profound in females [62].

A wealth of information regarding algal compounds and their anti-cellular senescence effects has been acquired. Some compounds exhibit anti-replicative senescence effect, whereas some exhibit anti-stress-induced senescence capability. The following are some examples.

Fucoidan is an algal compound that rescues cells from both replicative and stress-induced senescence. Lee et al. found that fucoidan reduced senescence in long-term cultured endothelial colony-forming cells, indicated by alleviation of the senescence-induced senescence-associated beta-galactosidase (SA-β-Gal) activity [63]. The SA- β-Gal activity is the most widely used biomarker for identifying senescent cells [64]. Moreover, fucoidan decreases pro-senescence protein p21 expression, but increases anti-senescence protein regucalcin expression [63]. Another study has reported that the treatment with fucoidan also reduces the SA-β-Gal activity induced by p-cresol, a major uremic toxin, in mesenchymal stem cells [65]. Moreover, specific compounds isolated from algae, such as porphyran, showed the anti-SA-β-Gal activity in stress-induced senescent fibroblasts [66].

In addition, phycoerythrin is a group of red photosynthetic pigments present in red algae. Synthesized phycoerythrin-derived peptide from the red alga *Pyropia yezoensis* downregulated the activity of SA-β-Gal in aged primary hippocampal neuron cells and attenuated age-dependent degeneration of neurites [67].

Although microalgae are not the focus of this review, some of them also exhibit the anti-aging potential. For example, *Chlorella vulgaris* is a green microalgae, and its hot water extract has been showed to increase human fibroblast population in the G0/G1 phase [68], which is a characteristic of senescence [69]. This hot water extract also exhibited protective effect against H_2_O_2_-induced oxidative stress and rescued length shortening of telomere in response to H_2_O_2_ treatment in cultured skin fibroblasts collected from human subjects [70]. As telomere shortening is a classic marker of cellular senescence [71], such telomere shortening preventive effect suggests the anti-stress-induced senescence effect of this microalga extract.

The anti-senescence capacity and lifespan extension effect of seaweed derivatives in lower organisms encourage studies on the anti-aging effect of algal compounds in higher animals. Although the effects of algal derivatives on major pathways that regulate lifespan have been reported, to the best of our knowledge, the lifespan extension effect of seaweed-derived bioactive compounds has not been evaluated using mammalian models. Herein, by discussing the anti-aging pathway regulation effects of bioactive compounds in seaweeds, based on both in vitro and in vivo studies, we hope to elucidate the potential anti-aging effect of bioactive compounds in seaweeds and explore the possibility of applying them in future studies.

## 3. Anti-Aging Pathway Regulation by Bioactive Compounds from Seaweeds

The major anti-aging related pathways identified include the SIRT, AMPK, autophagy, and IGF signaling pathways [72]. Other factors are also known to contribute to aging regulation, such as the antioxidant protein expression regulator and nuclear factor erythroid 2-related factor 2 (NRF2) [4]. However, these pathways are not independent of each other. They usually work in an integrative manner and regulate the onset or progress of aging via cross-talks among them.

### 3.1. AMPK and SIRT Pathways Activated by the Bioactive Compounds from Seaweeds

#### 3.1.1. AMPK and SIRT Pathways

Sirtuins (SIRT1–SIRT7) are a family of nicotinamide adenine dinucleotide (NAD+)-dependent protein deacetylases. SIRT1 shuttles between the nucleus and cytoplasm, whereas SIRT2 is localized in the cytoplasm only. SIRT3, SIRT4, and SIRT5 are mitochondrial proteins, whereas SIRT6 and SIRT7 reside in the nucleus and nucleolus, respectively [73]. Their downstream deacetylation targets include a wide range of signaling molecules, transcription factors, histones, and other enzymes [74]. SIRT1 is the mammalian ortholog most highly related to yeast SIR2, which is the most widely studied sirtuin [74]. Endogenous SIRT1 protein expression declines during replicative aging in human fibroblasts [75]. Even in mice, the expression of SIRT1 decreased with aging in organs such as the kidney and skin [76]. Its extra genetic copies can mimic the effects of calorie restriction and has been shown to extend the lifespan of multiple organisms, including yeasts, flies, and mice [77,78,79]. Furthermore, small molecule activators of SIRT1, such as resveratrol and SRT1720, also exhibit the lifespan extension ability in yeasts and mice [80,81]. There are several cellular senescence-related SIRT1 substrates, including, but not limited to, tumor repressor p53, autophagy negative regulator the mammalian target of rapamycin (mTOR), and cell growth-regulating transcription factors forkhead box protein O (FOXO) [82].

In addition to SIRT1, other sirtuins also modulate longevity and age-related diseases [73,82]. For example, mice with genetically overexpressed SIRT6 have a significantly longer lifespan than that of wild-type mice. However, such lifespan extension effect was observed only in male mice [83].

Besides SIRT, AMPK can also be activated by calorie restriction, and it has been proven to be essential for the lifespan extension effect with calorie restriction [84,85]. Both SIRT1 and AMPK are regulated by cellular energy levels. SIRT1 is activated by increased NAD+/NADH ratio, whereas stresses such as nutrient deprivation that raise the AMP/ATP ratio can activate AMPK [74]. Partially, SIRT1 prevents senescence by mediating the activation of AMPK through liver kinase B1 (LKB1). Simultaneously, AMPK enhances SIRT1 activity by increasing the cellular NAD+ level [86].

AMPK is maximally active when phosphorylated. As a serine/threonine protein kinase, by phosphorylating targeted metabolic enzymes and regulating related gene expressions, AMPK suppresses the synthesis of protein and fatty acid, but stimulates the oxidation of fatty acid and glycolysis to generate ATP [74]. AMPK regulates the aging process via an integrated signaling network. It can activate the signaling pathways such as FOXO, Nrf2, and p53; thus, improving the resistance of cells to stress [87].

Both total AMPK and phospho-AMPK expression levels decreased in the adipose tissue of aged mouse compared with those of its young counterpart [88]. Emerging evidence suggests that the responsiveness of AMPK signaling also declines with aging [87]. The lifespan extension function of AMPK has been observed in several studies. The transgenic expression of AMPK in adult fat body or adult muscle extended the lifespan of Drosophila, whereas reduced AMPK expression by RNA interference decreased their lifespan [84]. Additionally, metformin, an AMPK-activating molecule, improves both lifespan and health span in mice [89,90]. More noticeably, such health-beneficial effects have been observed in male mice even when the supplementation starts from middle age [89].

Obesity accelerates the aging of adipose tissue [91]. Adipose tissue from genetically obese mice showed features of premature aging, such as increased SA-β-Gal activity, p53 expression, and proinflammatory cytokines production [92]. The SIRT1 and AMPK pathways can also exert anti-aging effects in overweight subjects besides normal body weight subjects. The activities of both SIRT1 and AMPK increase with resveratrol supplementation in high fat diet-fed mice, increasing their survival [93]. Calorie restriction-induced activation of AMPK and SIRT1 has also been found in overweight male humans [94].

#### 3.1.2. SIRT and AMPK Activators from Seaweeds

Sirtuin-activating compounds have been studied for their anti-aging effects [80,81,95]. The most well-known sirtuin-activating compound is resveratrol, a natural phenol in grapes and berries [81]. Some bioactive compounds in seaweeds have displayed sirtuin-activation ability under various conditions (Table 1).

*Ecklonia cava* is an edible marine brown alga found in the coast of Japan and Korea. Its polyphenol extract has been reported to exhibit SIRT1- and AMPK-activation effects in high-fat induced obese mice. The polyphenol extract supplementation alleviated the decrease in hepatic SIRT1 protein level caused by high fat diet, and the level of hepatic phosphorylated AMPK and the expression of its downstream genes were significantly increased by this extract [96].

Another study reported that the methanol extract of *E. cava*, which is also rich in polyphenol, likewise showed the ability to increase the phosphorylation level of AMPK after incubation with C2C12 mouse myoblasts for only 1 h [97]. When this methanol extract was administered to mice with streptozotocin-induced type I diabetes, it alleviated their fasting glucose level and restored their plasma insulin concentration [97].

A meroterpenoid-rich faction of ethanol extract from another brown alga *Sargassum serratifolium* was found to increase the hepatic phospho-AMPK level in high fat-fed mice [98]. This fraction, which contains high levels of sargahydroquinoic acid, sargachromenol, and sargaquinoic acid, alleviated obesity and non-alcoholic fatty liver disease induced by high fat diet [98].

In addition, increased SIRT1 activity can be observed in conjunctival epithelial cells when incubated with the extract of brown alga *A. nodosum* [99].

Some red algae have also shown the SIRT1- and/or AMPK-activation capabilities. For instance, phenol-rich extract of *Gracilaria verrucosa*, a red alga, inhibited lipid accumulation in differentiated 3T3-L1 murine adipocytes [100]. Furthermore, *G. verrucosa* extract increased glucose uptake, and more importantly, AMPK phosphorylation during 3T3-L1 differentiation [100].

Besides the ethanol and methanol extracts of algae, which contain a mixture of various chemicals, specific compounds from different algae have also been reported to exhibit AMPK- and/or SIRT-activation activities.

Two chemicals isolated from *E. cava*, phloroglucinol and dieckol, have shown significant AMPK-enhancement ability [40,101,102]. Treating immortalized human hepatocytes HepG2 with phloroglucinol, a major phenolic compound in *E. cava*, increased the cellular phospho-AMPK levels [101]. Oral administration of phloroglucinol also significantly improved glucose tolerance in male mice that were fed a high fat diet [101]. Dieckol, a phlorotannin, is found in the brown algae *E. cava* and *Ecklonia stolonifera*. Treating 3T3-L1 adipocytes with dieckol during differentiation showed AMPK activation capability, leading to the inhibition of adipogenesis [40]. Such an AMPK-activating effect of dieckol has also been observed in *db/db* mice with type II diabetes. With intraperitoneal administration for 14 days, the phosphorylation level of AMPK was higher in the muscle tissues of the dieckol-administrated group than in the saline-treated control group. In addition, their blood glucose level, serum insulin level, and body weight were significantly reduced, compared with those of the control group [102].

Besides *E. cava*, bioactive compounds from other brown algae also exhibit AMPK- and/or SIRT-activation abilities. For instance, octaphlorethol A, another phlorotannin isolated from the brown alga *Ishige foliacea*, exhibited muscle AMPK-activation effects in *db/db* mice with type II diabetes [103]. Two weeks of intraperitoneal injection of octaphlorethol A showed beneficial effects on hyperglycemia in mice with diabetes. It significantly decreased the postprandial blood glucose level and the level continuously decreased until the end of the experiments, which was at week 5 [103].

Another study revealed that two indole derivatives, indole-2-carboxaldehyde and indole-5-6-carboxyaldehyde, isolated from *Sargassum thunbergii*, another kind of brown alga, showed AMPK-phosphorylation induction effect in a dose-dependent manner in differentiated 3T3-L1 adipocytes [104]. Their inhibitory effects on lipid accumulation and adipogenesis have been proposed to activate the AMPK signaling pathway [104].

Fucosterol, a sterol metabolite isolated from *Ecklonia stolonifera*, has been reported to enhance the expression of SIRT1 and suppress the phosphorylation of FOXO1 in 3T3-L1 [105]. Decreased phospho-FOXO1 expression indicated its deacetylation by elevated SIRT1 [115].

Fucoidan, whose main constituent is fucose, is found mainly in various species of brown algae. The size of fucoidan can vary from over 100 kDa to less than 500 Da [116]. Besides its effect on inducing phospho-AMPK in human hepatoma cells, fucoidan also showed the ability to increase phospho-AMPK and nuclear SIRT protein levels in streptozotocin-treated β cells. Its ability to ameliorate streptozotocin-induced pancreatic β cell death and impaired insulin synthesis has been suppressed by an SIRT1 inhibitor, indicating that fucoidan exerts such effects in a SIRT1-dependent manner [107]. Besides SIRT1, fucoidan also showed pronounced SIRT6 activation effect [108]. As SIRT6 has also been implicated in the regulation of cellular senescence [117,118,119], investigating the anti-aging effect of fucoidan seems appealing.

Low-molecular-weight fucoidan (LMWF) extracted from *Saccharina japonica* (molecular weight of 6500 Da) was found to protect the liver from injury in *db/db* mice with diabetes [109]. Administration of LMWF decreased the level of liver dysfunction markers, such as plasma alanine aminotransferase and aspartate aminotransferase as well as hepatic triglyceride and cholesterol. Compared with those of the control C57BL-C mice, *db/db* mice had low hepatic phospho-AMPK and SIRT1 levels. Supplementation of LWMF to *db/db* mice can increase the phospho-AMPK and SIRT1 levels. More importantly, with an SIRT1 inhibitor or AMPK inhibitor, the protective effect against oxidative stress and inflammation in HepG2 hepatic cells was almost completely reversed [109]. These findings suggest that LMWF protects the liver from injury through the SIRT1/AMPK pathways.

The AMPK activation effect of LMWF has also been reported by another study [110]. Low-molecular-weight fucoidan obtained by acid hydrolysis of fucoidan extracted from *Undaria pinnatifida* can increase the phospho-AMPK level in the skeletal muscle of *db/db* mice with diabetes. It also stimulated the expression of phospho-AMPK in L6 myotubes, leading to glucose uptake and fatty acid oxidation [110].

In addition to SIRT1, LMWF is also engaged in the activation of SIRT3, a mitochondrial sirtuin [111]. Treating aged mice with traumatic brain injury (TBI) with LMWF improved their long-term neurobehavioral outcomes. Both the mRNA and protein levels of neuronal SIRT3 decreased in normal aged mice compared with those in their young counterparts. The administration of LMWF elevated SIRT3 expression after TBI in aged mice. Knockdown of SIRT3 using intracerebroventricular injection of siRNA partially suppressed the neuroprotective effect of LMWF [111].

Fucoxanthin is another algal compound whose nutraceutical effects against obesity and cancer have been characterized [120,121]. It can increase the phosphorylation of AMPK as well as the expression of SIRT1 in oleic acid-induced hepatocytes FL83B cells [112]. The phosphorylation of AMPK elevated by fucoxanthin in both the skeletal muscle and liver of *db/db* mice suggests that fucoxanthin regulates the AMPK signaling pathway in the muscle and liver of *db/db* mice with type II diabetes [113]. This might contribute to the health benefits of fucoxanthin in these *db/db* mice, including alleviation of body weight gain and insulin resistance, as well as decrease in epididymal fat weight and fasting glucose level [113]. In another study, in response to oxidative stress trigged by arachidonic acid and iron, fucoxanthin induced the phosphorylation of AMPK in HepG2 cells and alleviated oxidative damage [114].

These studies suggest a promising role of seaweed bioactive compounds in anti-aging, at cellular and tissue levels. Most animal studies involving bioactive compounds in seaweeds and the SIRT or AMPK pathway have been conducted in obese or diabetic animals. However, whether these seaweed derivatives can stimulate the baseline SIRT and phospho-AMPK expression levels during aging has not been elucidated, especially in mammals. Thus, further research on whether the seaweed-derived bioactive compounds such as fucoidan and fucoxanthin can be used to extend the lifespan of higher organisms, such as rodents and primates, is needed.

### 3.2. Autophagy Activated by the Bioactive Compounds from Seaweeds

#### 3.2.1. Autophagy in Aging

There are three main types of autophagy, viz., macroautophagy, microautophagy, and chaperone-mediated autophagy. Macroautophagy (hereafter referred to as autophagy) is the major type that will be discussed here. Autophagy facilitates the degradation and recycling of unnecessary or dysfunctional intracellular components and thus is essential for cellular homeostasis [122].

The autophagy process is initiated by the engulfment of material that needs to be degraded by the phagophore. This double-membrane structure then elongates and sequesters the marked cellular components until it matures into an autophagosome, which travels through the cytoplasm to the lysosome and fuses with it to form an autolysosome [122]. Within the autolysosome, the contents and inner membrane of the autolysosome are degraded by the lysosomal enzymes. Autophagy-related (ATG) proteins play a central role in autophagy machinery construction [123]. For example, microtubule-associated protein 1 light chain 3 (LC3-I/ATG8) is conjugated to the lipid phosphatidylethanolamine by ATG7 and ATG3 to form LC3-II/ATG8-PE [122]. The converted LC3-II then promotes the formation and lengthening of the autophagosome and thus is essential for autophagosome biogenesis [124]. Beclin 1 (ATG6) is another autophagy effector, which along with other factors forms a complex contributing to the formation of autophagosome [125]. Besides ATG proteins, mTOR is a key inhibitor of autophagy whose activity can be activated by phosphorylation [126].

During normal and pathological aging, autophagy becomes insufficient, because of either diminished autophagic flux or excessive cargo resulting from chronic cellular damage [127]. The accumulation of dysfunctional cellular components might stimulate the cellular senescence process. Studies have shown that genetic inhibition or disruption of autophagy induces age-associated degenerative changes in mammalian tissues and decreases the lifespan of flies [128,129]. On the contrary, enhanced autophagy can delay aging [130]. Calorie restriction can induce autophagy; inhibition of autophagy prevents the anti-aging effect of calorie restriction in several species, such as *Caenorhabditis elegans*, *Drosophila melanogaster*, and mice [128]. Rapamycin is an autophagy inducer, whose long-term administration can extend the lifespan of mouse and flies [131]. Moreover, increase in lifespan has been observed when the autophagy gene *LC3* is overexpressed in the neurons of adult flies [132].

The autophagy process is not independent of the AMPK or SIRT pathway. The AMPK protein is an important upstream regulator of autophagy, which stimulates the autophagic processes by inhibiting mammalian target of rapamycin complex 1, a negative regulator of autophagy [128]. As the knockout or knockdown of Atg genes abolishes the lifespan prolonging effect of SIRT1, autophagy is also required for the lifespan-prolonging effect of SIRT1 [133].

#### 3.2.2. Autophagy Activators from Seaweeds

The effects of various bioactive compounds in seaweeds on autophagy have been studied [134,135,136,137,138,139,140]. However, studies on the induction or inhibition effect of different compounds on autophagy have shown inconsistent results. The differences are likely due to different experiment conditions. When study subjects are under stress, they may respond differently, compared with that under normal conditions. Interestingly, most studies have used specific compounds from seaweeds instead of crude extracts (Table 2).

Algal carotenoid fucoxanthin induced autophagy in gastric cancer SGC7901 cells, indicated by the up-regulated expression of Beclin-1 and LC3 [134]. In human epithelial cervical cancer Hela cells, fucoxanthin dose-dependently increased the expression of LC3-II and Beclin-1, and decreased the level of mTOR [135]. Fucoxanthin also exhibited neuroprotection in a mouse model of TBI by activating autophagy, indicated by increased expression of LC3-II and Beclin-1 [136]. In addition, in hepatocytes under oxidative stress, fucoxanthin increases the level of LC3-II and Beclin-1, and decreases the phosphorylation of mTOR [114,134]. Other algal-derived agents, including EPA and DHA, are also potent inducers of autophagy as indicated by the formation of autophagosomes in DHA- or EPA-treated lung adenocarcinoma A549 cells [137].

The AMPK- and SIRT-activation effects of fucoidan have been discussed earlier in this review. Effect of fucoidan on autophagy has also been studied. Fucoidan treatment increased the number of autophagosomes in human multiple myeloma U266 cells. The expression of LC3-II and Beclin-1 also increased, whereas the expression of phospho-mTOR decreased in fucoidan-treated cells [138].

In contrast, anti-autophagy effect of fucoidan in the liver has been observed under multiple pathological conditions, such as hepatic fibrosis and ischemia-reperfusion (I-R) [136,139]. Similarly, inhibition of autophagy by another algal compound fucosterol has also been observed in acutely injured liver. In response to concanavalin A-induced acute liver injury, pretreatment with fucosterol alleviated hepatic damage by inhibiting autophagy [140]. Interestingly, in normal animals without acute liver injury, fucosterol did not affect autophagy [140].

These anti-autophagy effects might not be totally unexpected due to the functions of autophagy in liver diseases. Autophagy is required for the activation of hepatic stellate cells, which produce extracellular matrix in the liver during fibrosis; therefore, the inhibition of autophagy can ameliorate liver fibrosis [141]. Liver autophagy exhibits both beneficial and detrimental effects following hepatic I-R and therefore the role of autophagy in hepatic I-R remains to be elucidated [142]. Overall, the functions of autophagy in liver diseases make it more complicated to study and interpret the influence of algal compounds, such as fucoidan under normal conditions.

### 3.3. Insulin/IGF-1 Signaling Pathway Inhibited by the Bioactive Compounds from Seaweeds

#### 3.3.1. Insulin/IGF-1 Pathway

Insulin is a blood glucose-regulating hormone secreted by the pancreas. Insulin-like growth factor 1 (IGF-1) is a hormone structurally similar to that of insulin. It is critical during childhood growth and has anabolic effects in adults [143].

Unlike the previously mentioned AMPK, SIRT, and autophagy pathways, the elevated insulin/IGF-1 pathway contributes to aging in several organisms [143]. Excess insulin signaling damages cellular function and accelerates aging. The insulin/IGF signaling pathway affects lifespan in several model organisms, including worms, flies, and mice. Mice with insulin receptor homozygous deletion specific to fat cells have extended lifespan in both sexes [144].

In mammals, insulin receptor (IR) and IGF-1 receptor (IGF-1R) have been identified as the major receptors for insulin and IGF-1, respectively [143]. The receptors are activated by their ligands, insulin and IGF-1. Ligand binding activates their intrinsic tyrosine kinase activity, resulting in autophosphorylation and activation of receptors [145]. The activated receptors transduce the signal to activate a sequential signaling cascade, leading to the phosphorylation and retention of the FOXO transcription factors in the cytoplasm. FOXOs play a critical role in the longevity regulation function of insulin/IGF-1 signaling [146]. Moreover, FOXO stimulates the transcription of genes related to stress resistance, growth, metabolism, and cellular differentiation [147]. Genetic variants of FOXO have been shown a significant association with human longevity in different ethnic groups [148,149,150,151]. Upon suppression of insulin/IGF-1 signaling, activated FOXOs might exert lifespan extension effect.

The lifespan extension effect of suppressed insulin/IGF-1 signaling has been shown in humans. However, compared to that of other key lifespan extension pathways discussed here, insulin/IGF-1 signaling shows a more profound sex difference in humans. Polymorphism analysis has shown that genetic variation causing reduced insulin/IGF-1 signaling activity is beneficial for old age survival in women, but not in men [5]. In another study of nonagenarians, female subjects with IGF-1 below the median levels presented significantly longer survival than that of female subjects with IGF-1 above the median levels. However, such a survival advantage was not been observed in males [152].

#### 3.3.2. Insulin/IGF-1 Inhibitors from Seaweeds

The insulin/IGF-1 signaling suppressors, along with FOXO activators, have been used as both algal crude extracts and isolated compounds (Table 3). Hot-water-soluble polysaccharide of the green alga *Capsosiphon fulvescens* significantly inhibited the phosphorylation of IGF-1R and insulin receptor substrate 1 in response to IGF-1 in AGS human gastric cancer cells. Insulin receptor substrate 1 is a key target of the insulin receptor, which can be phosphorylated by the activated insulin receptor [153]. The phosphorylation of Akt, a downstream target of insulin and IGF-1, was also inhibited [154].

In contrary, LMWF showed IGF-1 induction ability in mice with bladder cancer treated with chemotherapy drugs [155]. A combination of LMWF and chemotherapy drugs elevated IGF-1 expression and formation, and decreased FOXO3 expression and activation in mice compared with those in mice administered chemotherapy only [155]. Similar to autophagy regulation in pathologic liver, the effects of fucoidan on IGF-1/insulin under normal conditions need further investigation as the mice in this study were under severe pathological circumstances.

Furthermore, methanolic extract of *Chondrus crispus*, an edible strain of red alga, enhanced oxidative stress tolerance in in *Caenorhabditis elegans*. Noticeably, treatment with this methanolic extract increased the transcription of *daf16* gene, the sole ortholog of *FOXO* in the nematode *C. elegans* [156]. Moreover, kappa-carrageenan, a major component from *C. crispus* water extract has also been found to induce *daf16* activation in *C. elegans*, in response to pathogen infection (*Pseudomonas aeruginosa*). This is essential for immune response enhancement by kappa-carrageenan in the process [157]. In addition, a fucose-containing polymer-rich fraction from the brown alga *Ascophyllum nodosum* prolonged the lifespan in *C. elegans* at 20, 30, and 35 °C. The expression level of *daf16* was not altered by fucose-containing polymer treatment under control conditions (at 20 °C), whereas it increased significantly under heat-stressed conditions (at 30 and 35 °C) [158].

Similar to studies on AMPK and/or SIRT activation, elevated FOXO3 and daf16 levels by seaweed derivatives have been studied under stress conditions, such as oxidative stress, pathogen infection, and heat stress. However, whether these compounds can exert insulin/IGF-1 inhibition and FOXOs activation effects during aging need further research.

### 3.4. NRF2 Pathway Activated by the Bioactive Compounds from Seaweeds

#### 3.4.1. NRF2 Signaling Pathway

Besides the key pathways mentioned previously, antioxidant and anti-inflammatory effects are also considered to protect against aging. Excessive oxidative stress and low-grade chronic inflammation might contribute to the onset of aging [4]. Increased oxidative stress is a major characteristic of aging and implicated in various age-related pathologies. During aging, the production of oxidants increases, whereas the level of various antioxidant enzymes decreases. The excessive oxidative products cause cellular damage. NRF2 is a transcription factor whose signaling pathway mediates cellular responses to defend against oxidative stress. Kelch-like ECH-associated protein 1 (Keap1) is a specific repressor of NRF2. By interacting with Keap1, NRF2 remains in the cytoplasm. Acting as an NRF2-specific E3 ligase adaptor protein, Keap1 conjugation also promotes the degradation of NRF2 [159]. In the presence of oxidative stress or other stimuli, Nrf2 disassociates from Keap1 and translocates into the nucleus in a free and stabilized form. As a transcription factor, Nrf2 exerts its function within the nucleus. By dimerization with a small Maf protein, NRF2 activates the antioxidant response element (ARE), a DNA promoter located in the genes of several antioxidant proteins and detoxifying enzymes [160]. The Nrf2-ARE pathway can induce antioxidant enzymes such as superoxide dismutase (SOD) and catalase (CAT), as well as the detoxifying enzymes, including hemoxygenase1 (HO-1), glutathione S-transferase (GST), and NADPH:quinone oxidoreductase (NQO1) [160].

NRF2 is required for calorie restriction. NRF2 signaling protects against the sequelae of oxidative stress, including aging and aging-related diseases. Aging flies progressively lose their ability to activate NRF2 targets in response to acute stress exposure [161]. In humans, NRF2-ARE downstream gene activation, in response to stimuli, such as exercise, is impaired with aging [162]. Preserving NRF2 signaling competence can antagonize age-associated functional decline [161]. Keap1 loss-of-function mutations have been shown to extend the lifespan of Drosophila, supporting the longevity-regulating role of NRF2 [163]. A comparative study of lifespan among rodent species showed that constitutive NRF2-signaling activity (ARE-binding activity) is positively correlated with their lifespan [164]. Impaired activity of the NRF2 antioxidant pathway is a driver mechanism of Hutchinson–Gilford progeria syndrome (HGPS), a rare and invariably fatal premature aging disorder, whereas the reactivation of NRF2 can reverse cellular HGPS defects [165]. NRF2 also shows tissue-specific protective effects against aging. For example, the outer retina of Nrf2-deficient mice were more vulnerable to age-related macular degeneration than wild-type mice [166].

#### 3.4.2. NRF2 Activators from Seaweeds

Both extracts and fractions of certain seaweeds and specific compounds isolated from seaweeds have been reported to exhibit NRF2 activation effect (Table 4). Extracts and several fractions derived from cultivated green alga potently activated the NRF2-ARE pathway in IMR-32 neuroblastma and LNCaP prostate cancer cells [167]. The selected fractions induced nuclear translocation of NRF2 and transcription of *NQO-1*, a target gene of NRF2 [167].

Moreover, ethanol extract of *S. serratifolium*, a marine brown alga, induced NRF2 protein expression in RAW 264.7 macrophages. A downstream target, HO-1, increased its protein expression, whereas the Keap1 protein level was suppressed by the ethanol extract in a dose-dependent manner [168].

In addition, a phlorotannin-rich extract of another brown alga *E. cava* also induced the expression of NRF2 and HO-1, both in the absence and presence of lipopolysaccharide, in macrophages [169]. Lipopolysaccharides activate the membrane-bound NADPH oxidase in macrophages and promote oxidative stress.

Different algal compounds, from unsaturated fatty acids to polysaccharides, from sargaquinoic acid to carotenoids, have been shown to exhibit NRF2-activating capacities. From the green alga *Ulva lactuca*, Wang et al. extracted unsaturated fatty acid (C18:1(n-11)), which activated the NRF2/ARE pathway-regulated cytoprotective genes, including *NQO1* and *HO1* in human neuroblastoma IMR-32 cells. Analysis of various organs of mice, including the brain, heart, lung, liver, and stomach, further validated ARE-activation effect of *Ulva* extract enriched with C18:1(n–11) [170].

Polysaccharides from *Sargassum fusiforme*, a brown alga, activated antioxidant defense by promoting NRF2-dependent cytoprotection and ameliorated stress insult during aging in mice [171]. The cytoplasmic and nuclear Nrf-2 protein levels declined with aging in the liver of male mice. Two months after the administration of polysaccharide to middle-aged mice (9-month old) increased the total protein expression and nuclear accumulation of NRF2 in the liver of mice. The NQO1 protein level also increased simultaneously [171].

Treated Chinese Hamster lung fibroblasts with indole-6-carboxaldehyde (I6CA) isolated from *Sargassum thunbergii* prevented the oxidative-induced cell cycle arrest through activation of Nrf-2 signaling pathway. The protein expressions of Nrf-2 and HO-1, and the phosphorylation of Nrf-2 were greatly increased when the cells were treated with both H_2_O_2_ and I6CA, compared to the treatment of H_2_O_2_ alone [174].

Seaweed-produced mycosporine-like amino acid (MAAs) are water-soluble metabolites that absorb UV radiation [172]. Two MAAs, shinorine and porphyra-334, are competitive inhibitors of Keap1-NRF2 binding and have the potential to activate the NRF2-ARE pathway [172].

In addition, sargaquinoic acid isolated from the brown alga *Myagropsis myagroides*, showed anti-inflammatory activity upon LPS stimulation in RAW 264.7 cells [173]. The nucleus NRF2 and total HO-1 protein levels increased by sargaquinoic acid treatment in response to LPS [173].

Dieckol isolated from *Ecklonia stolonifera* induced the activation of NRF2 and increased the expression of NRF2 target proteins, including HO-1, NQO-1, and GST, in HepG2 cells via the NRF2/ARE pathway [27]. Another phlorotannin isolated from *E. stolonifera*, eckol, was also found to stimulate the nuclear translocation of NRF2 and induce the expression of HO-1 in HepG2 cells [28]. Zonarol, a para-hydroquinone-type pro-electrophilic compound from the brown alga *Dictyopteris undulata*, activated the NRF2/ARE pathway, inducing NRF2 target genes such as *HO-1* and *NQO1* in hippocampal neuronal HT22 cells. Moreover, zonarol increased the survival of HT22 cells in response to cell death-inducing glutamate. The neuroprotective function of zonarol can be attributed, at least in part, to the activated NRF2/ARE pathway [175]. Fucoxanthin also increased the accumulation of nuclear NRF2 and increased the transcription of *HO-1* and *NQO1* in mouse hepatocytes BNL CL.2 [176]. Various groups of seaweed derivatives exhibiting NRF2 activation effects strongly corroborate the antioxidant effects of algal compounds. Such an NRF2-stimulating effect also might exert anti-aging functions with other aging-regulating pathways.

## 4. Conclusions and Future Prospects

Increasing demand for natural products with health-promoting properties has increased the need for developing nutraceuticals, which are safe and have reliable natural origins. Although various macroalgae species across the world have been consumed as food and natural medicines for centuries, studies evaluating their possible anti-aging effect are limited.

This review summarizes the potential functions of seaweed extracts or compounds on aging-relating pathways. Regulation of these pathways suggests a promising role of macroalgal bioactive compounds in anti-aging, at both cellular and tissue levels; sometimes they also exhibit anti-obesity, anti-diabetes, and neuroprotective effects. Senescence is known to associate with multiple disorders, such as obesity, type II diabetes, and cognition declining. The protective effect of seaweed-derived bioactive compounds against these diseases also suggests the plausibility of utilizing them in anti-aging application. Studies on lifespan and health span extension effects of seaweed derivatives on aged animals are needed to bring insights into the functions of these compounds. Meanwhile, the influence of sex on anti-aging effects of these seaweed-derived substances should also be examined. As discussed herein, several compounds, such as meroterpenoid and fucoxanthin, have shown the ability to regulate multiple anti-aging pathways. These compounds need more attention, and it is appealing to explore their anti-aging effects, whether they can provide protective effect against age-related diseases, such as cardiovascular and neurodegenerative diseases, and whether they could extend lifespans, especially in higher animal models.

## Figures and Tables

**Table 1:** Bioactive compounds in seaweeds showing activation on sirtuin and/or AMP-activated protein kinase.

Source	Active Component	Major Activity	Reference
*Ecklonia cava*	Polyphenol extract	Increase the hepatic phospho-AMPK and SIRT1 protein expressions in high-fat induced obese mice	[96]
*Ecklonia cava*	Methanol extract	Increase the phosphorylation level of AMPK after incubation with C2C12 mouse myoblast cells	[97]
*Sargassum serratifolium*	Meroterpenoid-rich fraction	Increase the hepatic phospho-AMPK level in high fat fed mice	[98]
*Ascophyllum nodosum*	Extract	Increase nuclear SIRT1 activity in conjunctival epithelial cells	[99]
*Gracilaria verrucosa*	Phenol rich ethanol extract	Elevate cellular phospho-AMPK expression in differentiated 3T3-L1 adipocytes	[100]
*Ecklonia cava*	phloroglucinol	Increase cellular phospho-AMPK levels in HepG2 immortalized human hepatocytes	[101]
*Ecklonia cava or E. stolonifera*	Dieckol	Elevate phospho-AMPK level in muscle tissue of db/db mice with type II diabetes	[102]
		Activate AMPK pathway in differentiated 3T3-L1 adipocytes	[40]
*Ishige foliacea*	Octaphlorethol A	Enhance the muscle phospho-AMPK levels on db/db mice with type II diabetes	[103]
*Sargassum thunbergii*	Indole-2-carboxaldehyde and indole-5-6-carboxyaldehyde	Activate AMPK pathway in differentiated 3T3-L1 adipocytes	[104]
*Ecklonia stolonifera*	Fucosterol	Enhance SIRT1 expression in 3T3-L1 adipocytes	[105]
Not specified	Fucoidan	Induce AMPK phosphorylation in poorly differentiated human hepatoma cells	[106]
		Increase phosphor-AMPK and nuclear SIRT protein levels in streptozotocin (STZ)-treated β cells	[107]
		Enhance SIRT6 deacetylation activity ex vitro	[108]
*Sacharina japonica*	Low-molecular-weight fucoidan	Elevate hepatic SIRT1 and phospho-AMPK levels in db/db mice	[109]
*Ubdaria pinnatifida*	Low-molecular-weight fucoidan	Increase the phospho-AMPK level in L6 myotubes and skeletal muscle of diabetic db/db mice	[110]
Not specified	Low-molecular-weight fucoidan	Enhance neuronal SIRT3 levels in aged mice with traumatic brain injury	[111]
Not specified	Fucoxanthin	Increase the expression of phospho-AMPK and SIRT1 in oleic acid-induced hepatocytes FL83B cells	[112]
		Increase the AMPK phosphorylation levels in both skeletal muscle and liver of db/db mice	[113]
		Elevate the phospho-AMPK level in human liver carcinoma HepG2 cells	[114]

**Table 2:** Bioactive compounds in seaweeds showing regulation on autophagy.

Source	Active Component	Major Activity	Reference
Not specified	Fucoxanthin	Upregulate Beclin-1 and LC-3 protein levels in gastric cancer SGC7901 cells	[134]
		Increase the levels of Beclin-1 and LC-3 proteins and decrease the level of mTOR in human epithelial cervical cancer Hela cells	[135]
		Elevate Beclin-1 and LC-3 protein expressions in traumatic mouse brain	[136]
		Increase the protein levels of LC3-II and Beclin-1, and decrease the phosphorylation of mTOR in hepatocytes under oxidative stress	[114]
Not specified	EPA and DHA	Enhance the formation of autophasosomes in lung adenocarcinoma A459 cells	[137]
Not specified	fucoidan	Increase the formation of autophasosomes and LC3-II and Beclin-1 protein levels, decreased phospho-mTOR level in human multiple myeloma U266 cells	[138]
		Inhibit the autophagosome formation and decrease the LC3 and Beclin-1 protein levels in hepatic fibrosis	[136]
*Fucus vesiculosus*	fucoidan	Downregulate Beclin-1 and LC3 expressions in hepatic ischemia-reperfusion	[139]
	fucosterol	Decrease the Beclin-1 and LC3 protein levels in acute liver injury	[140]

**Table 3:** Bioactive compounds in seaweeds with insulin/IGF-1 inhibition activity.

Source	Active Component	Major Activity	Reference
*Capsosiphon fulvescens*	Hot-water-soluble polysaccharide	Inhibit the phosphorylation levels of IGF-1R and IRS-1 in response to IGF-1 in AGS human gastric cancer cells	[154]
Not specified	Low-molecular-weight fucoidan (LMWF)	Combination of LMWF and chemotherapy drugs elevates IGF-1 expression and formation, and decreases FOXO3 expression and activation in mice with bladder cancer	[155]
*Chondrus crispus*	methanolic extract	Increase daf16 gene transcription in *C. aenorhabditis elegans*	[156]
*Chondrus crispus*	kappa-carrageenan	Induce daf16 mRNA level in response to pathogen infection in *C. elegans*	[157]
*Ascophyllum nodosum*	a fucose containing polymer-rich fraction	Increase daf16 transcription under heat-stress conditions	[158]

**Table 4:** Bioactive compounds in seaweeds with NRF2 activation activity.

Source	Active Component	Major Activity	Reference
Cultivated green alga	Extracts and certain fractions	Induce NRF2 nuclear translocation and transcription of *NQO-1* in IMR-32 neuroblastma and LNCaP prostate cancer cells	[167]
*Sargassum serratifolium*	Ethanol extract	Induce NRF2 and HO-1 protein expressions and suppress KEAP1 protein expression in RAW 264.7 macrophages	[168]
*Ecklonia cava*	phlorotannin-rich extract	Induce the protein expression levels of NRF2 and HO-1, in the absence or in the presence of LPS, in macrophages	[169]
*Ulva lactuca*	unsaturated fatty acid (C18:1(n-11))	Induce transcription of *NQO1*, and *HO1* in human neuroblastoma IMR-32 cells and mice brain, heart, lung, liver, and stomach	[170]
*Sargassum fusiforme*	polysaccharides	Increase total protein expression and nuclear accumulation of NRF2 in middle aged mouse liver	[171]
*Sargassum thunbergii*	Indole-6-Carboxaldehyde	Increase the expression and phosphorylation of Nrf-2 in the presence of H_2_O_2_	
Not specified	shinorine and porphyra-334	Display a competitive inhibiting activity of Keap1-NRF2 binding	[172]
*Myagropsis myagroides*	Sargaquinoic acid	Increase nucleus NRF2 protein levels in macrophages response to LPS	[173]
